# Treatment and Prevention of Postoperative Leakage after Gastrectomy for Gastric Cancer

**DOI:** 10.3390/jcm12123880

**Published:** 2023-06-06

**Authors:** Sang-Ho Jeong, Jin-Kwon Lee, Kyung Won Seo, Jae-Seok Min

**Affiliations:** 1 Department of Surgery, Gyeongsang National University School of Medicine & Gyoengsang National University Changwon Hospital, Changwon 51471, Republic of Korea; jshgnu@gmail.com (S.-H.J.); gsbigcap@gmail.com (J.-K.L.); 2 Department of Surgery, Kosin University Gospel Hospital, Busan 49267, Republic of Korea; hahachristi@gmail.com; 3 Department of Surgery, Dongnam Institute of Radiological and Medical Sciences, Cancer Center, Busan 46033, Republic of Korea

**Keywords:** anastomotic leakage, surgical procedures, operative, gastrectomy, stomach neoplasm

## Abstract

Anastomotic leakage is one of the common causes of serious morbidity and death after gastrectomy. The use of surgical treatment for leakage decreased due to the development of nonsurgical management. However, if nonsurgical management fails to control the spread of intra-abdominal infection, emergency surgical treatment is required. The authors wished to determine in which cases surgical treatment is needed for postoperative leakage and to identify treatment and prevention strategies. If a patient’s vital signs are stable, local abscesses can be cured by conservative treatment after percutaneous drain insertion; if there is no improvement in anastomotic leakage, endoscopic treatment such as clipping, vacuum, and stent placement can be performed. If a patient’s vital signs are unstable or patient shows diffuse peritonitis, surgical treatment should be performed. A surgical plan can be established according to leakage location. The duodenal stump may first require conservative treatment. It is recommended that surgical treatment be attempted first for anastomotic leakage of gastrojejunostomy site and gastric stump in remnant stomach. In conclusion, the need for surgical treatment is determined depending on vital signs and presence of diffuse peritonitis. During surgical treatment, a strategic approach is required according to the patient’s condition and the anatomical location of leakage.

## 1. Introduction

Gastric cancer is one of the most common cancers worldwide. According to the data from GLOBOCAN 2020 that investigated the incidence and mortality of cancers, the percentage of new cases and deaths from gastric cancer was 5.6% (5th place) and 7.7% (4th place) among all cancers in 2020 [1]. Gastric cancer still remains a malignant disease of great concern around the world. The recent increase in the incidence of gastric cancer among young adults worldwide in the past decade is noteworthy [1]. For the treatment of gastric cancer, endoscopic submucosal dissection can be considered as the first modality in cases of EGC with mucosal confinement, well or moderately differentiated tubular adenocarcinoma or papillary carcinoma, a clinical tumor size of >2 cm, and without evidence of ulceration in the tumor by endoscopy, or size of EGC tumor ≤3 cm, mucosal cancer, and an ulcer in the tumor by endoscopy [2,3]. Endoscopic resection can result in a better quality of life, shorter hospital stay, and lower incidence of complications after treatment compared to gastrectomy [4,5,6,7]. It is also recommended to perform curative radical gastrectomy if endoscopic submucosal dissection is not applicable. Laparoscopic distal gastrectomy is recommended for early staged gastric cancer located in the lower stomach [3].

The goal of treatment for gastric cancer is the complete removal of tumors without any residual gastric malignancy. Various treatment methods for gastric cancer are briefly summarized as follows [8]. Endoscopic resection was performed as an alternative to gastrectomy for patients with a minimal risk of lymph node metastasis, particularly in cases of small-sized early gastric cancer (EGC) [3,9,10]. If endoscopic resection is inappropriate, gastrectomy is recommended for resectable cases. The principle of gastrectomy for gastric cancer involves adequate lymph node dissection, sufficient resection margins, and functional anastomosis. Laparoscopic surgery is often recommended for distal gastrectomy in cases involving EGC. Function-preserving surgery, which includes pylorus-preserving or proximal gastrectomy, was recommended in selected cases of EGC. For resectable advanced gastric cancer, open surgery for gastrectomy is still often recommended, although this was not definitively confirmed [3,9]. In cases of distant metastatic gastric cancer, palliative gastrectomy can be considered to manage intractable symptoms or life-threatening situations.

The incidence of complications after gastrectomy for gastric cancer is approximately 19.9 to 40%, and the 30-day mortality rate after gastrectomy ranges from 3.4 to 5.4% [11,12]. One French study reported the incidence of postoperative leak is 7.1% (12/169), and other German study showed 7.5% (6/80) after total gastrectomy [13,14]. Recently, the incidence of early gastric cancer (EGC) gradually increased, and the use of minimally invasive surgery, such as laparoscopic and robotic surgery, also increased [15,16,17,18]. According to the data reported by the Korean Gastric Cancer Association in 2021, in South Korea, the overall complication rate was 14.5% and the mortality rate was only 1% in 2019. Additionally, the incidence of anastomotic leakage and duodenal stump leakage was 1.2% and 0.7%, respectively, which did not reach the current rate of 2%, showing a decrease compared to previous reports [16]. As a reason for this decrease, it is predicted that the development of energy devices reduced organ injury complications due to thermal damage, and the development of the stapler used during anastomosis reduced the incidence of intestinal anastomosis leakage [19,20,21,22,23,24]. In addition, the need for emergency reoperation decreased because of improvements in drugs, including antibiotics, and endoscopic and intervention techniques as treatments for complications after gastrectomy [25,26,27,28,29,30]. Nevertheless, emergency surgical treatment, rather than conservative or interventional treatments, is needed in critical cases. Gastrointestinal anastomotic leakage is one of the common causes of serious morbidity and death after surgery [11,31,32]. Therefore, in this review, we aimed to investigate the causes, diagnosis, and treatment of postoperative leakage after gastrectomy for gastric cancer. Additionally, the authors wished to determine in which cases surgical treatment is needed for postoperative leakage and to identify treatment and prevention strategies when surgical treatment is necessary.

## 2. Causes of Postoperative Leakage

The causes of postoperative gastrointestinal leakage after gastrectomy for gastric cancer are as follows: poor circulation of blood around the gastrointestinal anastomotic site, excessive tension at the anastomotic site, high intraluminal pressure at the anastomotic site due to stenosis or obstruction at the distal low viscus, technical stapling failure (misfiring of the stapler), and suture failure (weak strength of the anastomotic site) [33,34,35,36,37,38,39]. 

## 3. Clinical Manifestation and Diagnosis of Postoperative Leakage

**Figure 1:**
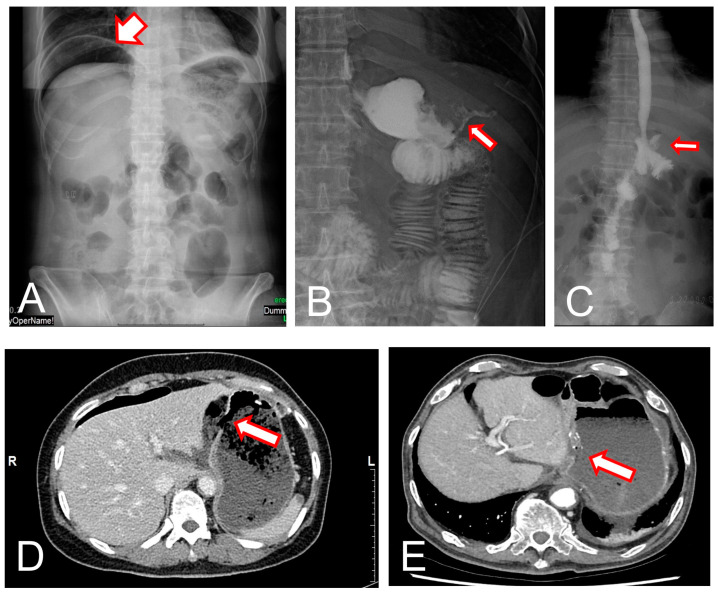
(**A**), Subphrenic pneumoperitoneum (arrow) that developed suddenly during the recovery period after surgery in simple abdomen X-ray. (**B**) Postoperative leak (arrow) showed in gastrojejunostomy site by upper gastrointestinal series. (**C**) Leak (arrow) showed in esophagojejunostomy site. (**D**,**E**) Defect (arrow) in the remnant stomach and food material around the stomach shown on an abdominal CT scan.

Postoperative gastrointestinal anastomotic leakage can arise from any site with sutures or staple lines, even including the jejunojejunostomy site of a Roux-en-Y anastomosis [40,41]. Most cases of anastomotic leakage commonly occur within the first 5 to 10 days after surgery [11,12,32]. Most patients present with fever, unexplained tachycardia, hypotension, abdominal pain, and/or an acute abdomen [12]. A subphrenic pneumoperitoneum developed suddenly during the recovery period after surgery in simple abdomen X-ray (chest PA or abdomen erect) (Figure 1A). When an anastomotic leak is suspected, a computed tomography (CT) scan, which may show indirect evidence of a leak, such as pneumoperitoneum, extraluminal contrast, inflammatory stranding, fluid collection, and/or abscesses, should be performed to diagnose leakage (Figure 1D,E) [42,43]. Contrast studies, such as an upper gastrointestinal series with gastrografin, may show the site of leakage (Figure 1B,C) [44,45]. Leakage can also be diagnosed with endoscopy directly; however, endoscopic diagnosis should be chosen carefully because leakage may worsen due to air insufflation during endoscopy [12,28,46,47]. 

## 4. Basic Treatment Strategy for Postoperative Leakage

**Figure 2:**
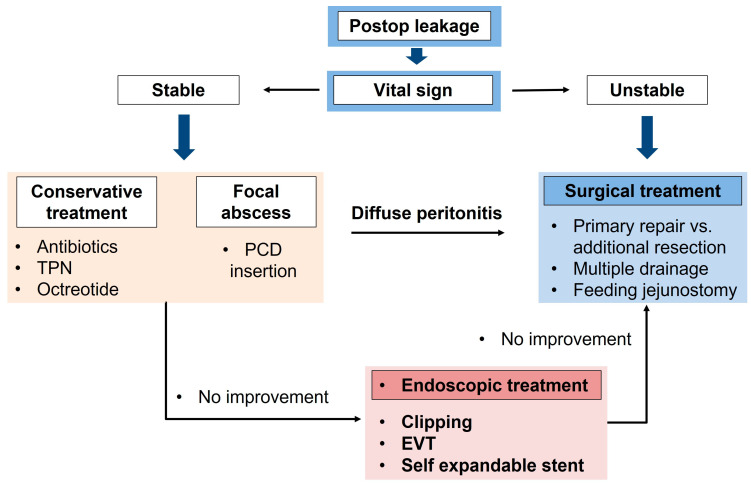
Treatment algorithm for anastomotic leakage after gastrectomy for gastric cancer. TPN, total parenteral nutrition; PCD, percutaneous catheter drainage; EVT, endoscopic vacuum therapy.

The general treatment principle for postoperative leakage are as follows [12,48] (Figure 2):

1. Broad-spectrum antibiotic infusions should be initially conducted;
2. Conservative treatment comprises intravenous fluid infusion therapy with nutritional support such as total parenteral nutrition [49,50];
3. Sometimes, a specific drug, such as somatostatin, is administered in the hope of reducing intestinal endocrine secretion, especially in cases of high output leakage or peritoneal drainage [35,51,52,53,54];
4. Further invasive management for the leak should be considered depending on the situation and condition of the patient, and interventional management could be provided by performing a percutaneous drainage procedure [55,56,57];
5. Intestinal intraluminal stenting is one option for invasive management [28,30]. If nonoperative interventions do not induce successful results for leakage management, the patient has an unstable hemodynamic status, or peritonitis occurs in the whole abdomen due to leakage, the patient should undergo an emergency operation;
6. Surgeons should perform exploratory abdominal surgery, and irrigation, drainage, and closure of the leakage site or revision of the anastomosis to treat and prevent worsening abdominal infection [28,58].

## 5. Endoscopic Treatment of Postoperative Leakage

The treatment of postoperative leakage using endoscopic techniques can be categorized into sealant application, OTSC, stenting, and endoscopic vacuum-assisted closure 5–24. Let us explore each approach in detail: endoscopic treatment has limitations depending on the size of the dehiscence (<2 cm or <70% of the circumference).

1. Sealant and fibrin glue can be sprayed or injected using an endoscope after the formation of the fistula tract, rather than immediately after the leakage occurs. According to Rbabgo et al., a closure rate of approximately 86.6% without complications was reported [59].
2. Endosuturing also showed its utility in managing chronic fistulae. Similar instruments such as over-the-scope clips (OTSC) demonstrated positive effects on gastrocutaneous fistulae and gastric leaks in several studies [58,59,60,61]. According to a systematic review, a 100% success rate was reported when applying leakage within one week, but <60% over time [62,63].
3. Endoscopic stenting is a viable treatment option for leaks at esophagojejunostomy sites following gastrectomy or prosthetic gastrectomy [64,65]. Puli et al. [66] conducted a meta-analysis on self-expandable metallic stents (SEMS) and self-expanding plastic stents (SEPS) and reported a leak closure rate of approximately 87.8% when the stents were removed 4 to 8 weeks after insertion. It should be noted that partially covered SEMS carry a risk of tissue in-growth complicating the removal process [67].
4. EVAC (endoscopic vacuum-assisted closure) was initially introduced for anastomotic leaks after rectal surgery and was also applied after upper gastrointestinal (UGI) surgery [68]. Recent studies showed its effectiveness, particularly in esophageal cancer, and ongoing attempts are being made to explore its use after gastric cancer surgery [69,70].

To summarize, endoscopic interventions offer diverse options for managing postoperative leakage, leading to effective closure and improved patient outcomes.

## 6. Factors Associated with Postoperative Leakage

1. Location of leakage site: Anastomotic leakage vs. duodenal stump leakage

**Figure 3:**
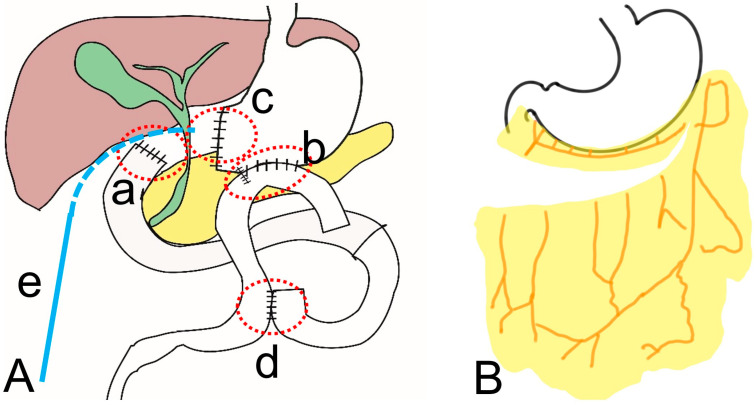
Factors associated with postoperative leakage. (**A**) Location of leakage at duodenal stump (**a**), gastrojejunostomy site (**b**), gastric stump (**c**), jejunojejunostomy site (**d**) placement of drainage catheter (**e**); (**B**) schematic figure of the remaining omentum.

Surgical treatment is required more often in cases of anastomotic leakage than in cases of duodenal stump leakage [71,72]. In Figure 3A, looking at the location of the leakage, the duodenal stump (a) is mostly trapped under the hepatic area (subhepatic space). Therefore, it is highly likely to cause only localized peritonitis in the subhepatic space. However, when leakage occurs away from the subhepatic space, mainly on the left side of the abdomen, such as at the gastrojejunostomy site (b), gastric stump (c), or jejunojejunostomy site (d), the leakage discharge can flow into the abdominal cavity like free perforation and causes generalized peritonitis in many cases.

Recently, authors reported on reoperation data after gastrectomy for gastric cancer. We reported that among the 1568 patients who underwent gastrectomy from 2009 to 2018, anastomotic leakage occurred in 2.02% (*n* = 31) of the patients, and duodenal stump leakage occurred in 1.17% (*n* = 18) of the patients [73]. Among these patients, the reoperation rate was 70% (*n* = 22/31) for anastomosis leakage patients and 5.5% (*n* = 1/18) for duodenal stump leakage patients; consequently, the reoperation rate for anastomosis leakage patients was significantly higher. Most of the duodenal stump leakage patients, that is, 94.5%, were cured with conservative management alone. A possible reason why anastomotic leakage has a higher reoperation rate than duodenal stump leakage is because anastomotic leakage causes generalized peritonitis rather than localized peritonitis. The expected reasons for this are as follows.

2. Existence of surgical drainage or percutaneous drainage

If an intra-abdominal surgical drainage is inserted in an appropriate place around the leakage site, the symptoms may not be severe, or mild localized peritonitis may occur [74,75,76,77]. Even in the case of duodenal stump leakage (Figure 3A(a)), if the discharge is adequately removed by drainage catheter (Figure 3A(e)), the occurrence of generalized peritonitis can be prevented. In the case of anastomotic leakage (Figure 3A(b,d), generalized peritonitis may not occur in some cases if an appropriate intraperitoneal drainage catheters are inserted.

3. Remaining omentum

The incidence of EGC is increasing, especially in the East [16,78]. In these cases, partial omentectomy is mostly performed during gastrectomy for EGC; consequently, the number of partial omentectomies is increasing [9]. The remaining omentum surrounds the leakage site, reducing the possibility of generalized peritonitis. Therefore, if there is a higher risk of leakage, the remaining omentum can be used as a barrier by selectively performing partial omentectomy rather than total omentectomy (Figure 3B).

## 7. Surgical Treatment Strategy in Patients with Postoperative Leakage

1. Basic surgical treatment strategy
  ① If possible, try to save the stomach. The leakage rate is higher in esophagojejunostomy than in gastrojejunostomy [79,80].
  ② If possible, it is better to perform minimally invasive surgery. In the case of an open gastrectomy, the possibility of complications after surgery is usually higher compared to that of laparoscopic gastrectomy [81,82].
  ③ Recommendation of feeding jejunostomy during reoperation: when performing the reoperation, the recurrence rate of leakage is relatively high due to peritoneal inflammation; therefore, feeding jejunostomy can help patients’ recover by enteral tube feeding [83,84].
  ④ Insertion of multiple surgical drains in the dependent position during reoperation: when performing the reoperation, sufficient drainage of discharge is important even in cases with the recurrence of leakage.
2. Indication for emergency surgical treatment in postoperative leakage patients [35,36,38]
  ① Diffuse peritonitis signs with the abrupt onset of peritonitis symptoms
    - Bursting of the anastomotic site;
    - Expectation of persistent peritonitis due to food content spillage.
  ② Gastrojejunostomy site leakage is more likely to require reoperation than duodenal stump leakage

In the case of duodenal stump leakage (Figure 3A(a)), since the leakage site is located in the subhepatic area, it can be sealed near the liver, and severe peritonitis symptoms do not appear in many cases. However, in the case of gastrojejunostomy or jejunojejunostomy site leakage (Figure 3A(d)), there are few organs nearby to seal the leak; therefore, generalized peritonitis often occurs. If the omentum continues to be a sufficient potential leakage barrier after partial omentectomy, there is a possibility that the symptoms will improve by sealing the leak near the remaining omentum (Figure 3B).

  ③ Total dehiscence is more likely to need reoperation than partial dehiscence at the esophagojejunostomy anastomotic site

Partial ruptures are more likely to be cured by conservative treatment. Recently, the treatment method of applying suction using an endoscope together with a sponge on a Levin tube was used [85,86,87,88]. However, since the total dehiscence is distant and there is no possibility of improvement over time, reoperation surgery is required immediately.

**Figure 4:**
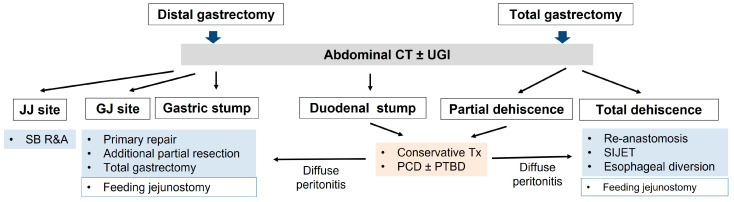
Surgical treatment plan by leakage location, CT, computed tomography; JJ, jejunojejunostomy; GJ, gastrojejunostomy; SB R&A, small bowel resection and anastomosis; PCD, percutaneous catheter drainage; PTBD, percutaneous transhepatic biliary drainage in this figure. Surgical treatment plan by leakage location, CT, computed tomography; JJ, jejunojejunostomy; GJ, gastrojejunostomy; SB R&A, small bowel resection and anastomosis; PCD, percutaneous catheter drainage; PTBD, percutaneous transhepatic biliary drainage; SIJET, suction isoperistaltic jejuno-esophagostomy tube.

Reoperation Strategy Based on Leakage Location (Figure 4) [35,38].

3. Surgical treatment after distal gastrectomy
  ① Rupturing of the stapler common entry hole at the gastrojejunostomy site (Figure 3A(b)): In general, when there is rupturing around the gastrojejunostomy site, the edema or inflammation in the surrounding tissues is severe, and so, proper debridement should be performed before closure. If the inflammation is severe, a new gastrojejunostomy is recommended after resection of the previous anastomosis. If the remnant stomach is too small, total gastrectomy should be performed if necessary.
  ② Leakage at the gastric stump site (Figure 3A(c)): In most cases, ischemic changes due to a decrease in blood supply are likely to be the cause of gastric stump leakage. Therefore, it is recommended to perform reresection of the gastric stump at the remnant stomach.
  ③ Leakage at the jejunojejunostomy site (Figure 3A(d)): In most cases, inflammation occurred, so it is necessary to perform resection and jejunojejunostomy reanastomosis rather than primary repair, which can cause leakage again after reoperation. Additional procedures recommended during reoperation are feeding jejunostomy and the insertion of multiple surgical drains.
4. Surgical treatment for complete dehiscence of the esophagojejunostomy site after total gastrectomy
  ① Re-anastomosis with feeding jejunostomy

**Figure 5:**
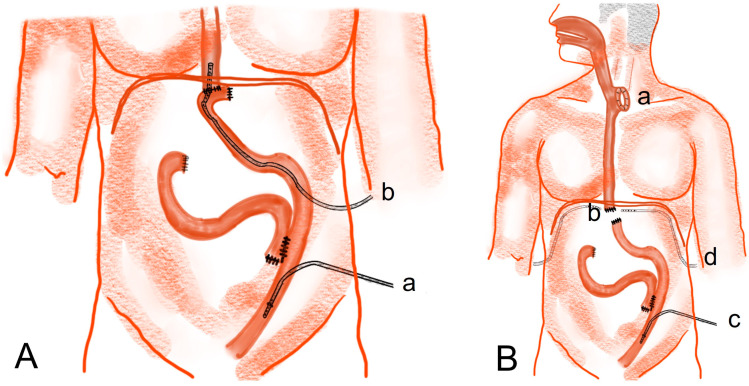
Esophagojejunostomy complete dehiscence surgical treatment. (**A**) Insertion of a continuous suction isoperistaltic jejuno-esophagostomy tube ((**a**), SIJET) with feeding jejunostomy (**b**) (**B**) Esophageal diversion (**a**), clamping of the distal esophagus (**b**), feeding jejunostomy (**c**) with multiple surgical drain tube (**d**).

Reanastomosis can be tried if the patient’s general condition is good. In the case of reoperation, the risk of releak is high, and so, feeding jejunostomy is recommended (Figure 5A(a)). In addition, multiple surgical drain tubes are essential.

  ② Insertion of a continuous suction isoperistaltic jejuno-esophagostomy tube (SIJET)

In a recently reported study, the authors reported successful recovery in two patients by inserting an 18F chest tube using gastroscopy during laparoscopic surgery (Figure 5A(b)) [89,90,91].

  ③ Esophageal diversion and clamping of the distal esophagus with feeding jejunostomy (esophageal exclusion)

This method is commonly used in esophageal perforation, and patients with infection due to esophageal perforation are subjected to esophageal exclusion surgery (esophagostomy and distal esophageal stapling, feeding jejunostomy) (Figure 5B) [92,93,94].

If the patient is in a septic condition due to severe inflammation, is elderly, or has many underlying diseases, esophageal diversion (Figure 5B(a)) may be considered.

**Figure 6:**
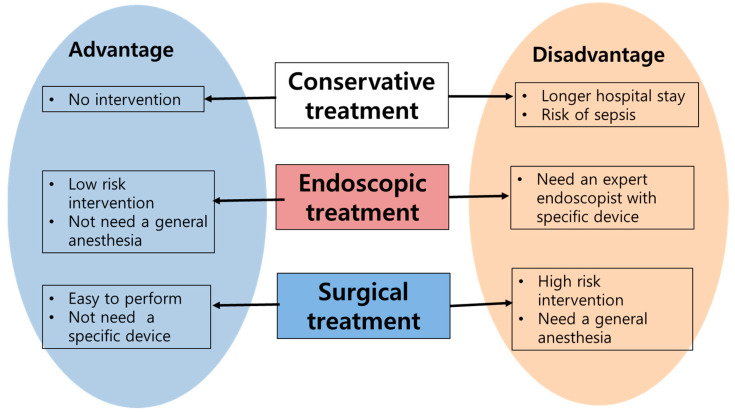
Three treatments for post-operative leakage has its own advantages and disadvantages.

**Three treatments for post-operative leakage has its own advantages and disadvantages (** Figure 6 **).**

1. Conservative treatment: This approach does not require surgical intervention, which could be considered a major benefit. However, the treatment period tends to be long. There is also a risk of sepsis, particularly in cases of severe peritonitis.

2. Endoscopic treatment: This is a low-risk procedure that does not require general anesthesia, making it a preferable option for some patients. However, there is a possibility of procedural failure. Moreover, the L-tube or stent used during the procedure often needs to be removed due to inadequate post-procedure management, which can necessitate multiple procedures. In addition, the use of special devices such as stents and vacuum, as well as the need for expert endoscopists, can limit the feasibility of this treatment option.

3. Surgical treatment: This treatment option can often be performed without the need for special equipment, which simplifies the process. However, there is a high likelihood of the patient requiring ventilator support and intensive care unit (ICU) admission due to postoperative pain.

## 8. Basic Strategy for the Prevention of Postoperative Leakage

**Figure 7:**
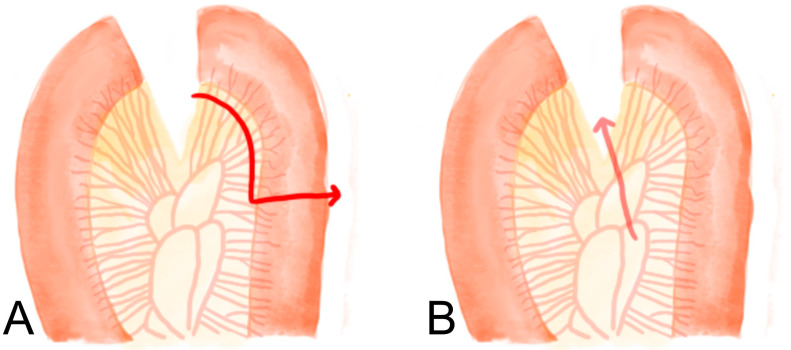
(**A**). Preservation of the mesentery vessel around the Roux limb (red arrow, cutting direction). (**B**) Reduction in tension at the anastomotic site (red arrow, cutting direction).

1. If the patient is thought to be malnourished before surgery, nutritional support is required [23,95].
2. Intraoperative air leak tests and endoscopic examinations during surgery are useful [96,97,98].
3. Preservation of sufficient blood circulation: Resection of the remnant stomach with proper blood supply is needed. In cases of total gastrectomy, the preservation of mesentery vessels around the Roux limb is essential (Figure 7A).
4. Reduction in tension at the anastomotic site: If the length of the Roux limp is increased by cutting the mesentery vessel, the tension at the anastomotic site can be reduced (Figure 7B).
5. Stapling failure: When dissecting a stomach or jejunum with a stapler, it is important to choose a stapler with an appropriate size and height. When dissecting very thick tissues or organs, the largest stapler should be selected [34].
6. Failure of suturing: When suturing using threads at the common channel of the stapler entry site, breakdown of the suture material may occur. In the case of barbed suture threads, the thickness and strength are reduced compared to those of general threads, so it is better to select a thread that is thicker than the general thread. The author of this paper commonly uses a barbed thread to close the stapler common entry hole at the gastrojejunostomy site. Previously, the author performed single-layer suturing using 3-0 barbed thread, but the author recently implemented double-layer suturing using 2-0 barbed thread.
7. Perioperative oxygen supply: There was a significant decrease in leakage in patients who received sufficient oxygen supplementation immediately after surgery [33,37].
8. Prevention of gastric stasis: If gastric stasis occurs after distal gastrectomy, an increase in pressure around the anastomosis can affect the occurrence of leakage at the anastomotic site. Therefore, an anastomosis method that does not cause gastric stasis should be selected. In addition, the diet protocol changes as the patient are taught to eat a small amount or dominant liquid diet immediately after gastrectomy.

**Figure 8:**
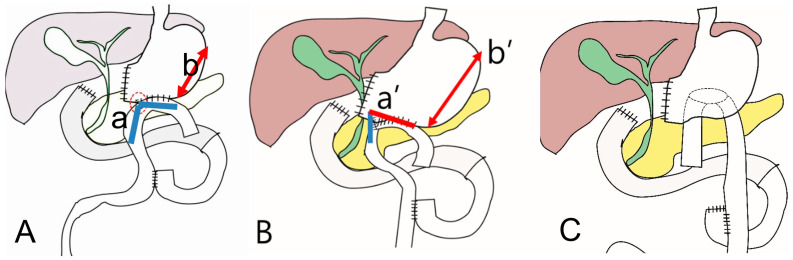
Prevention of post-operative leakage (**A**). Roux en Y gastro-jejunostomy with isoperistaltic anastomosis with pre-diet state, ((**a**), a blunt angle, (**b**) short greater curvature), (**B**). Roux en Y gastro-jejunostomy with food retention in the remnant stomach ((**a’**), an acute angle in gastrojejunostomy, (**b’**) longer greater curvature) (**C**). Roux en Y gastro-jejunostomy anti-peristaltic anastomosis with posterior wall anastomosis.

When performing gastro-jejunostomy with isoperistaltic anastomosis (Figure 7A), food retention in the remnant stomach can occur. In this case, as the greater curvature expands and the angle of the efferent loop of the gastro-jejunostomy changes a blunt angle (Figure 8A(a)) to an acute angle (Figure 8B(a’)), stenosis or obstruction may occur in the efferent loop. If food retention occurs in this way, symptoms such as vomiting can occur, and in severe cases, procedures such as endoscopic stent insertion or reoperation may be required. To prevent this mechanism, anti-peristaltic anastomosis is recommended (Figure 8C). In addition, if gastric stasis is a concern, it is helpful to conduct the gastro-jejunostomy anastomosis at the posterior wall of the remnant stomach, a little closer to the esophago-gastric junction rather than the greater curvature. In patients who have difficulty in postoperative ambulation due to old age or poor general condition, delayed gastric emptying may occur due to decreased motility of the remnant stomach, even if there is no structural obstruction at the gastro-jejunostomy anastomosis site. In these cases, it can be prevented by reducing the distance between the esophago-jejunostomy and gastro-jejunostomy sites.

## 9. Conclusions

The need for surgical treatment is determined depending on vital signs and the presence of diffuse peritonitis. During surgical treatment, a strategic approach is required according to the patient’s condition and the anatomical location of leakage.

## Data Availability

Not applicable.
